# Supplementary material for: A proline switch explains kinetic heterogeneity in a coupled folding and binding reaction
Source: Nat Commun. 2018 Aug 20;9:3332. doi: 10.1038/s41467-018-05725-0 (PMC6102232; doi:10.1038/s41467-018-05725-0)
Supplement: Supplementary file 1 — Supplementary Information [file 41467_2018_5725_MOESM1_ESM.pdf]

Supplementary Information for

A proline switch explains kinetic heterogeneity in a coupled folding and binding reaction

Zosel et al.

Supplementary Figures

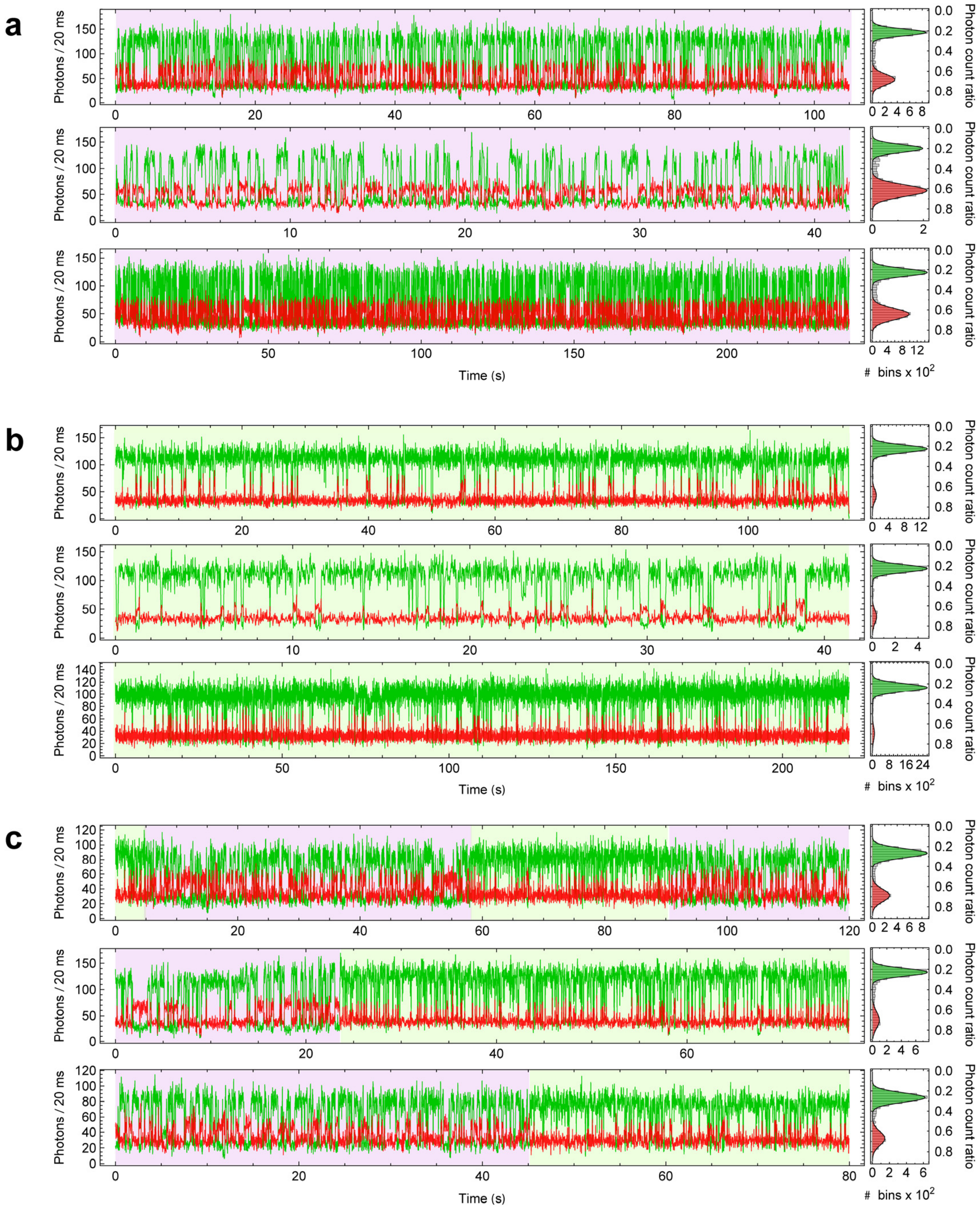

Supplementary Figure 1. Representative time traces of acceptor-labeled ACTR binding to immobilized, donor-labeled NCBD. Green: Donor photons; red: acceptor photons. The photon count ratio histograms (acceptor over total number of photons) of the traces are displayed to the right. Time traces were analyzed with the Viterbi algorithm using the model from Fig. 1e. (**a**) Examples of time traces where NCBD is in the high-affinity state for the whole time. (**b**) Examples of time traces where NCBD is in the low-affinity state for the whole time. (**c**) Examples of time traces where NCBD switches between high- and low-affinity state, with the high- and low-affinity segments (as identified by the Viterbi algorithm) shaded in light purple and light green, respectively.

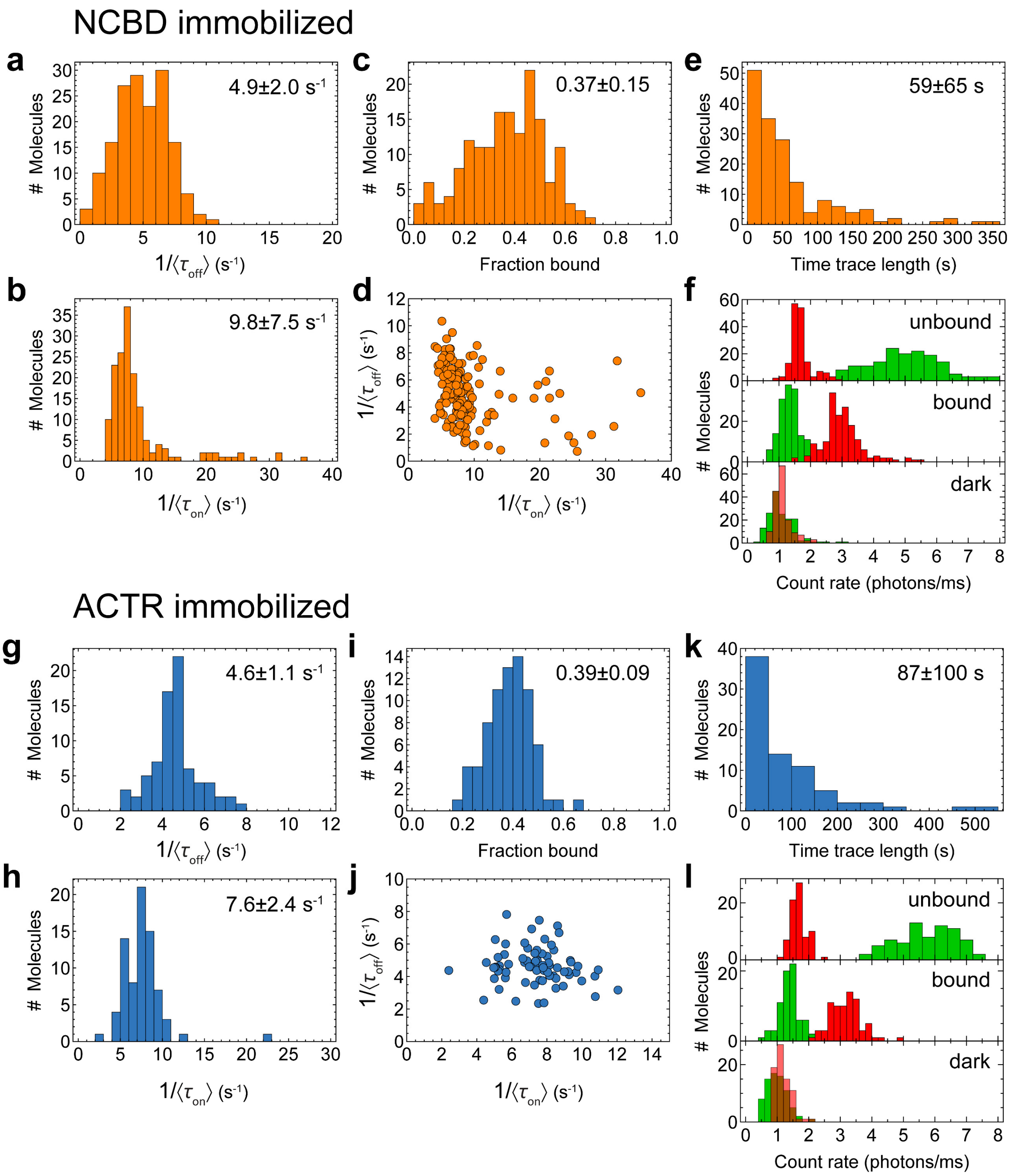

Supplementary Figure 2. Molecule-to molecule variability of immobilized NCBD and ACTR. Each event in the histograms corresponds to a single NCBD or ACTR molecule. Means and standard deviations of the displayed parameters are given. (**a**-**f**) Immobilized NCBD (cf. Fig. 1). (**a**) k'_{on} estimated from the reciprocal mean time spent in the unbound state, $1/\langle\tau_{off}\rangle$. (**b**) k_{off} estimated from the reciprocal mean time spent in the bound state, $1/\langle\tau_{on}\rangle$. The two subpopulations of NCBD (*cis*/*trans*) give rise to two clusters, around 7 and 30 s^{-1}. Molecules that switch between the two subpopulations within a time trace appear with intermediate $1/\langle\tau_{on}\rangle$. (**c**) Fraction of time that molecules spend in the bound state, calculated from $\sum\tau_{on}/\text{total time}$. (**d**) Scatter plot of $1/\langle\tau_{on}\rangle$ and $1/\langle\tau_{off}\rangle$. Molecules

with a large $1/\langle\tau_{\mathrm{on}}\rangle$ tend to have a smaller $1/\langle\tau_{\mathrm{off}}\rangle$, in accordance to the individual rate coefficients (Table 1). (**e**) Length of time traces until photobleaching. (**f**) Photon detection rates in the donor (green) and acceptor (red) channels, in the unbound (*upper panel*) and ACTR-bound state (*middle panel*), as well as in the dark state (*lower panel*). (**g-l**) Same as above, but for the experiment recorded with immobilized ACTR (cf. Fig. 2). The histograms of $1/\langle\tau_{\mathrm{off}}\rangle$, $1/\langle\tau_{\mathrm{on}}\rangle$ and the fraction bound are more homogeneous than for immobilized NCBD, since both NCBD subpopulations bind to ACTR randomly and their binding events are thus uncorrelated.

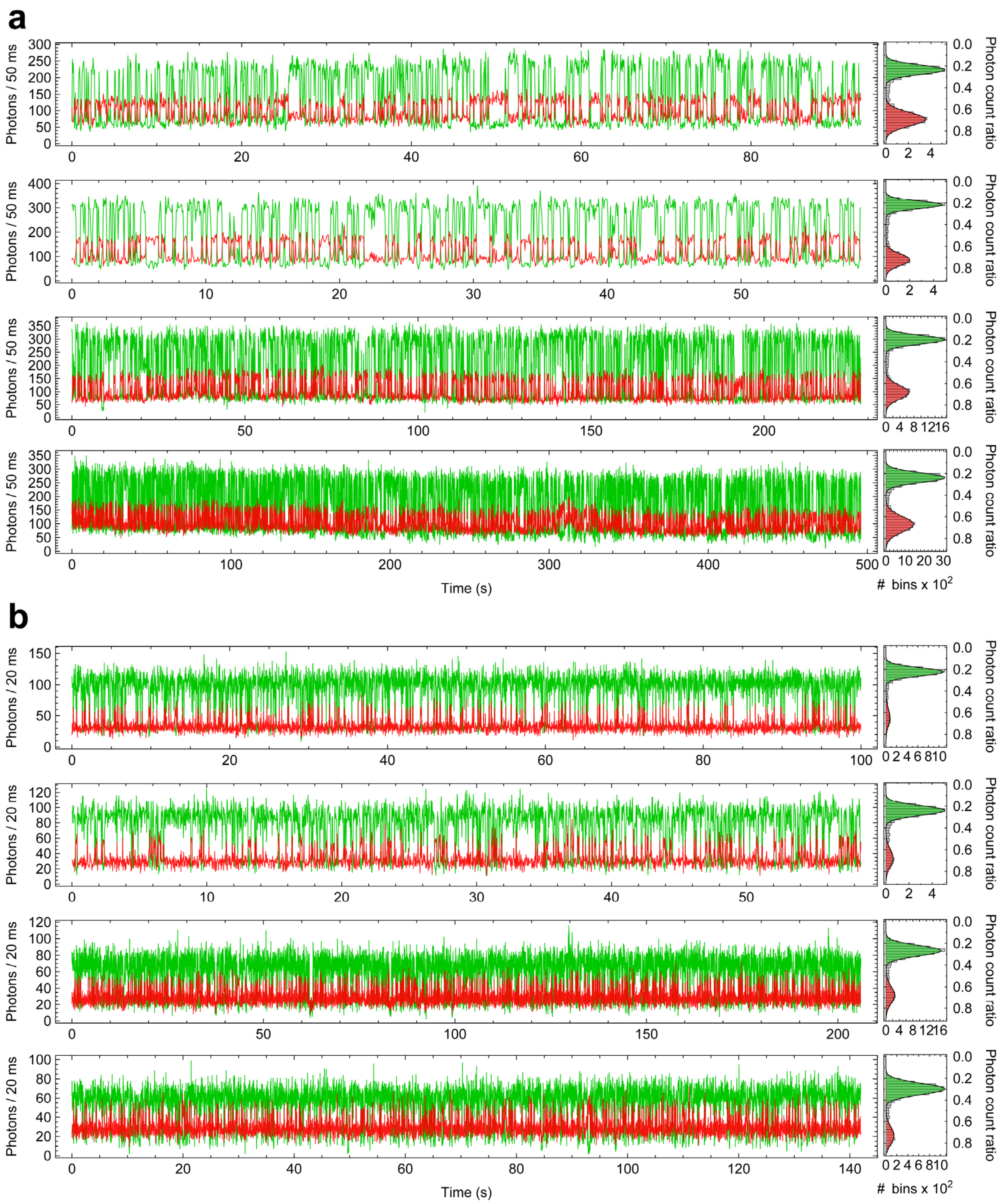

Supplementary Figure 3. Representative time traces. Green: Donor photons; red: acceptor photons. The photon count ratio histograms (acceptor over total number of photons) of the traces are displayed to the right. (**a**) Examples of time traces of NCBD binding to immobilized ACTR. (**b**) Examples of time traces of ACTR binding to immobilized NCBD P20A.

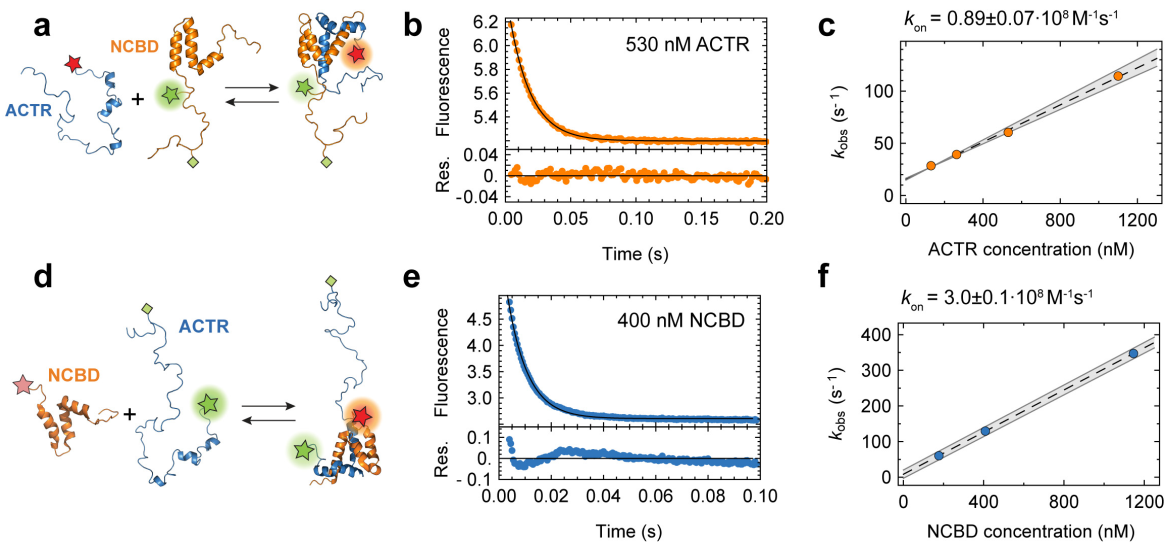

Supplementary Figure 4. Immobilization has only a small influence on the association rate coefficient. (**a**) Schematic representation of the reaction probed in the stopped-flow experiment. The same constructs as in the experiments with surface-immobilized molecules are used, but Cy3B-labeled, biotinylated NCBD remains free in solution. An excess of ACTR-CF680R is mixed with NCBD; the loss of Cy3B fluorescence reports on the formation of the NCBD-ACTR complex. (**b**) *Upper panel*: Average of five stopped-flow traces recorded at 530 nM ACTR, fitted with a single-exponential decay (black line). *Lower panel*: Residuals of the fit. (**c**) Association rate coefficients from stopped-flow experiment fitted with $k_{\mathrm{obs}} = k_{\mathrm{on,2state}} \cdot c_{\mathrm{NCBD}} + k_{\mathrm{off}}$ (dashed line). Even though this two-state simplification does not reflect the true binding mechanism, it suffices to estimate the overall association rate coefficient, k_{on} . The largest error arises from the uncertainty in ACTR concentration, due to adsorption of the protein to the syringe and tubing (the gray-shaded bands and errors given correspond to the uncertainty estimated from the difference in protein concentrations determined before and after the stopped-flow measurement). (**d-f**) Same as (**a-c**), but for binding of CF680R-labeled NCBD to Cy3B-labeled, biotinylated ACTR.

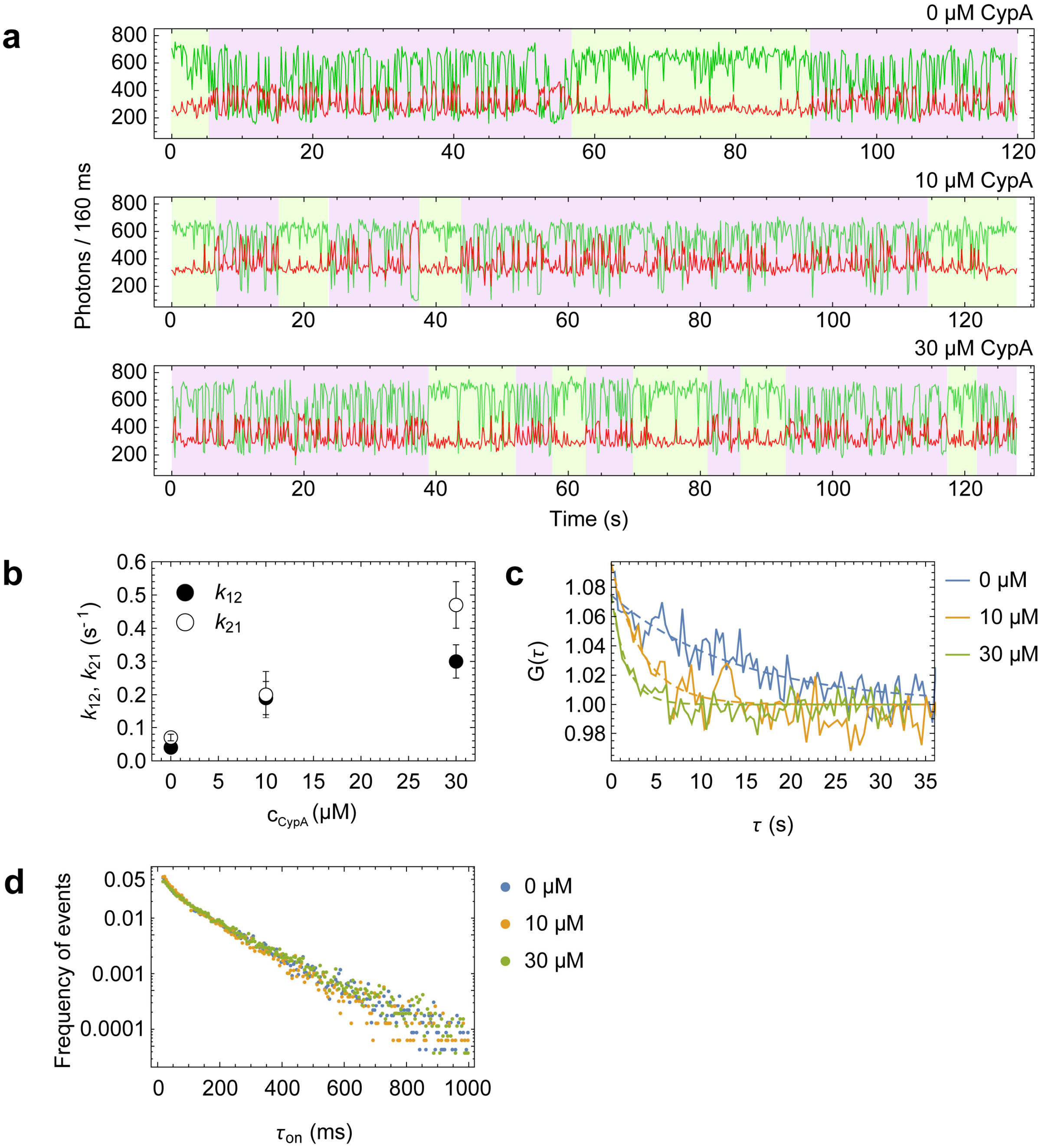

Supplementary Figure 5. Influence of Cyclophilin A (CypA) on the kinetics of ACTR binding to NCBD. (**a**) Representative time traces of CF680R-labeled ACTR binding to immobilized, Cy3B-labeled NCBD in the presence of CypA at the concentrations indicated. Green: donor emission; red: acceptor emission. The low-affinity segments (as identified by the Viterbi algorithm using the model from Fig. 1*E*) are shaded in light green, the high-affinity segments in light purple. (**b**) k_{12} and k_{21} as a function of CypA concentration determined by MLH analysis. (**c**) The correlation of the duration of consecutive binding events ($G(\tau)$, solid line) provides additional evidence that CypA accelerates switching between the two kinetic regimes. $G(\tau)$ was calculated for all time traces containing > 80 binding events and averaged. A value of $G(\tau) > 1$ indicates clustering of binding events with similar duration (long in the

high-affinity NCBD1 state, short in the low-affinity NCBD2 state). The decay of $G(\tau)$ reports on the exchange rate coefficient, k_{ex}, between the clusters. With increasing CypA concentration, $G(\tau)$ decays faster. The decay rate coefficients of single-exponential fits to $G(\tau)$ (dashed lines) are shown in Supplementary Table 1. (**d**) Overlay of the normalized bound-state dwell-time histograms in the absence and presence of CypA. The histograms are almost identical, indicating that CypA has no effect on the dissociation kinetics.

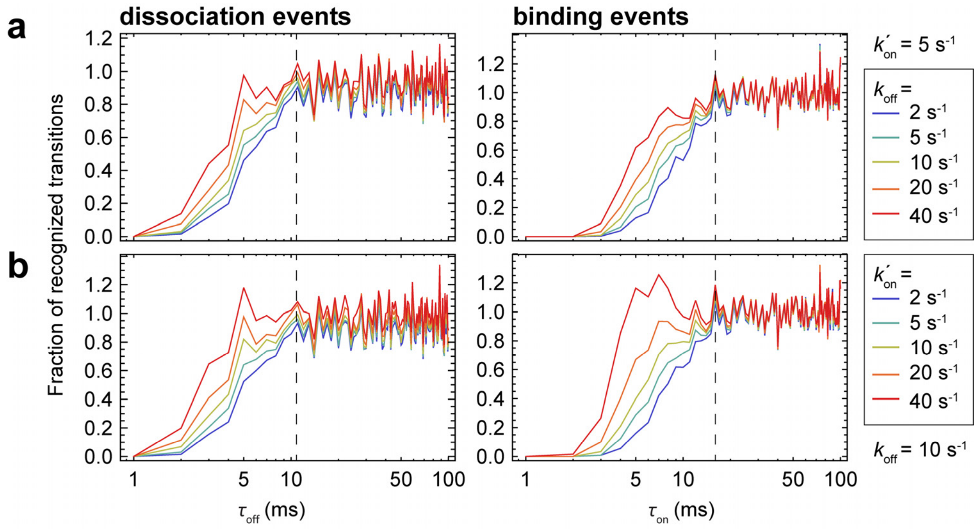

Supplementary Figure 6. Performance of the Viterbi algorithm for detecting short events. 162 single-molecule time traces were simulated based on the rate matrix $\mathbf{K}_{4\text{state,blink}}$ and the best-fit parameters from Table 1 (see Methods). Photon rates and durations of the time traces were taken from the measurements (see Supplementary Fig. 2). The simulated data were analyzed with the Viterbi algorithm using the rate matrix $\mathbf{K}_{2\text{state,blink}}$, which distinguishes between an unbound and a bound state, as well as a dark state that arises from donor blinking. Plotted is the fraction of recognized events, calculated by dividing the dwell time histogram of the identified trajectory by the dwell-time histogram of the original simulated state trajectory. Different guess values for k'_{on} and k_{off} were used, as indicated in the legend on the right. (**a**) guess value for k_{off} was varied; (**b**) guess value for k'_{on} was varied. Binding events are identified reliably for τ_{on} >16 ms; dissociation events for τ_{off} >11 ms (dashed lines). Taking the different photon count rates in both states into account, ~70 photons have to be detected for an event to be recognized correctly. Increasing the guess values for k'_{on} and k_{off} facilitates the identification of shorter events but has no influence on the identification of longer events. In all cases, the Viterbi algorithm recognizes >89% of all events; that fraction increases to 92% if values of k'_{on} = 5 s^{-1} and k_{off} = 10 s^{-1} are used, which are closest to the simulation input.

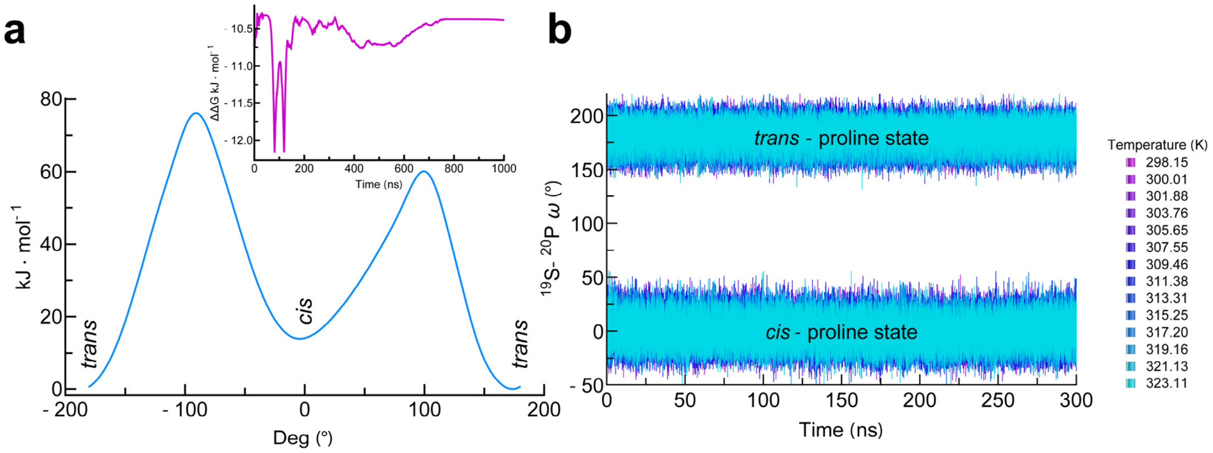

Supplementary Figure 7. Free energy surface of *cis/trans* isomerization of Ser19-Pro20 obtained from WT-metadynamics and ω-angle values sampled in REMD simulations. (**a**) Free energy profile of the *cis* and *trans* states of Pro20 integrated after 1 µs WT-metadynamics simulations. The inset shows the free-energy difference between the wells defining the *cis* (between -50 and 50 degrees) and the *trans* states as a function of the simulated time. After 700 ns, the energy between the states is not subject to variations anymore, indicating that the simulation has converged. (**b**) ω-angle as a function of time in the *trans* or *cis* states of Pro20 in REMD at the different temperatures chosen for each replica.

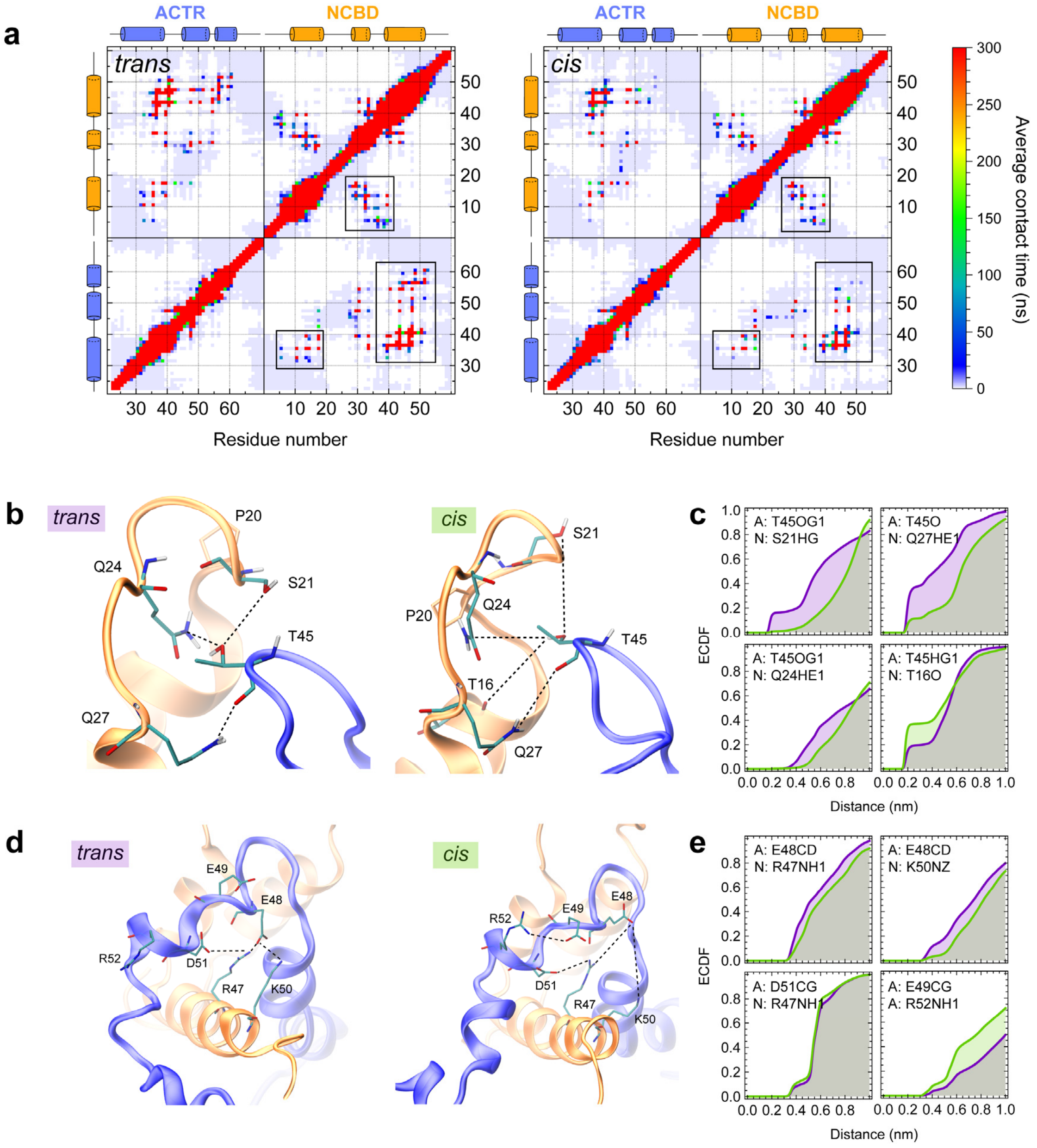

Supplementary Figure 8. Analysis of interactions within in the NCBD-ACTR complex from the MD simulations. (**a**) Diagonalized contact maps colored according to the average contact time of two residues falling within a distance cutoff of 0.65 nm. The secondary structure elements of ACTR and NCBD (according to the NMR structure, PDB code 1KBH[1]) are indicated next to the matrix. The black boxes highlight parts of the contact map with noticeable differences between the NCBD Pro20 *trans* and *cis* complexes. The maps indicate that the reduced stability mostly arises from the loss of intermolecular contacts. (**b-e**) Representative conformations of ACTR (blue) and NCBD (orange), showing which interactions are perturbed when NCBD Pro20 changes its isomerization state from *trans* to *cis*. Residues are shown using a stick representation, with atoms colored by atom type (carbon – cyan, oxygen – red, nitrogen – blue and polar hydrogen atoms – white). Figures were created using VMD and rendered using the Tachyon ray tracer built into VMD[2]. In (**b**), the interactions involving the loop connecting helices α1 and α2 of NCBD are depicted. The loop shifts its binding register in the *cis* state upon losing some interactions that occur in the *trans* state. Particularly, ACTR residue T45, which is involved in a series of hydrogen bonds with S21, Q24 and Q27 of NCBD, loses these interactions and establishes, in *cis*, a hydrogen bond with T16. The corresponding empirical distribution functions

(*trans*: purple, *cis*: green) are depicted in (**c**). In (**d**), interactions toward the C-terminal end of the molecules are shown, with the corresponding empirical distribution functions (*trans*: purple, *cis*: green) depicted in (**e**). The network of salt bridges rearranges in the *cis* state, with intermolecular interactions (ACTR E48–NCBD R47 and K50) being weakened, and intramolecular salt bridges (ACTR E49–R52) strengthened instead. Notably, the interaction between ACTR D51 and NCBD R47, which falls in the same region and was indicated as a crucial determinant of the NCBD-ACTR complex stability before[1], does not appear to be affected by the *cis*/*trans* switch.

...RSISPSALQDLLRTLKSPSSPQQQQVLNILKSNPQLMAAFIKQRTAKYVANQPG...

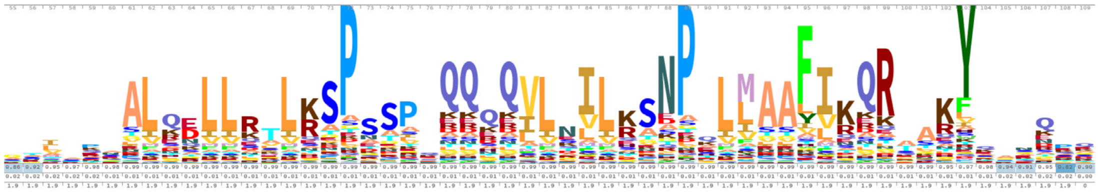

Supplementary Figure 9. Sequence conservation in NCBD. Relative amino acid probabilities at each position are shown as HMM logo[3], based on the analysis of 236 Creb-binding (PF09030) sequences in the Pfam database[4]. The sequence of the NCBD construct used here is shown on top.

Supplementary Tables

Supplementary Table 1. Addition of the peptidyl-prolyl *cis/trans* isomerase Cyclophilin A (CypA) accelerates interconversion between NCBD populations. Kinetic parameters of ACTR binding to surface-immobilized NCBD were obtained with MLH analysis, according to the kinetic model in Fig. 1*E*. $k_{off,1}$ and $k_{off,2}$ were constrained to the values in the absence of CypA, since the dwell-time histograms of the bound state are virtually identical under all conditions (cf. Supplementary Fig. 5d). k_{ex} reports on the decay of the correlation of the duration of consecutive binding events (cf. Supplementary Fig. 5c).

**[CypA]** (μM)	$k_{on,1} \cdot c_{ACTR}$ (s^{-1})	$k_{on,2} \cdot c_{ACTR}$ (s^{-1})	$k_{off,1}$ (s^{-1})	$k_{off,2}$ (s^{-1})	c_{ACTR} (nM) [a]	$k_{on,1}$ (10^8 $M^{-1}s^{-1}$)[b]	$k_{on,2}$ (10^8 $M^{-1}s^{-1}$)[b]	k_{12} (s^{-1})	k_{21} (s^{-1})	k_{ex} (s^{-1})[c]
0	6.0±0.2	3.0±0.5	7.3±0.3	30±3	65	0.93±0.06	0.46±0.08	0.04±0.01	0.07±0.01	0.07±0.02
10	4.4±0.1	2.2±0.2	7.3[d]	30[d]	61	0.72±0.04	0.36±0.04	0.19±0.05	0.20±0.07	0.29±0.09
30	6.2±0.2	2.6±0.3	7.3[d]	30[d]	72	0.86±0.05	0.36±0.05	0.30±0.05	0.47±0.07	0.58±0.14

Errors are the standard deviation based on ten bootstrapping trials if not stated otherwise.

[a] Concentrations of acceptor-labeled ACTR were determined using FCS (see Methods). An uncertainty of ±5% was estimated from two independent measurements conducted before and after recording the single-molecule time traces.

[b] Uncertainty from propagating the error of k'_{on} and the ACTR concentration.

[c] Determined by correlating the duration of consecutive binding events (Supplementary Fig. 5c).

[d] $k_{off,1}$ and $k_{off,2}$ were constrained to the values in the absence of CypA.

Supplementary Table 2. Table of all kinetic parameters of NCBD variants binding to surface-immobilized ACTR, determined with MLH analysis.

**variant**	$k_{on,1}\cdot c_{NCBD}$ (s^{-1})	$k_{on,2}\cdot c_{NCBD}$ (s^{-1})	$k_{off,1}$ (s^{-1})	$k_{off,2}$ (s^{-1})	c_{NCBD} **(nM)** [a]	$k_{on,tot}$ (10^8 $M^{-1}s^{-1}$)	***# events*** [c]
wild type	3.3±0.3	1.6±0.3	5.6±0.5	30±6	17	2.9±0.2	34852
P23A	5.2±0.2	1.5±0.1	8.3±0.3	48±2	25	2.7±0.2 [b]	44380
P7/37A	2.6±0.1	0.89±0.10	7.4±0.2	55±5	19	1.8±0.1 [b]	31939
P7/23/37A	2.1±0.1	0.81±0.08	11±1	62±10	13	2.2±0.1 [b]	32058
P20A	4.3±0.2	n.a.	23±1	n.a.	14	3.2±0.2	24294
P20/23A	4.0±0.1	n.a.	25±1	n.a.	16	2.5±0.1	30348
P7/20/23A	5.8±0.2	n.a.	19±1	n.a.	26	2.2±0.2	31617
P20/23/37A	5.7±0.2	n.a.	33±1	n.a.	23	2.4±0.2	21841
P7/20/23/37A	6.8±0.1	n.a.	28±2	n.a.	32	2.1±0.1	35533

n.a. not applicable
Errors are the standard deviation based on ten bootstrapping trials if not stated otherwise.
[a] Concentrations of acceptor-labeled NCBD were determined using FCS (see Methods). An uncertainty of ±5% was estimated from two independent measurements conducted before and after recording the single-molecule time traces.
[b] For bi-exponential decays, the sum $k'_{on,1}+k'_{on,2}$ was used to calculate $k_{on,tot}$.
[c] Sum of all detected binding and dissociation events.

Supplementary Table 3. Amino acid sequences of all protein variants used (after proteolytic cleavage of the His-Tag). The proline residue where the residue numbering of NCBD starts (position 1) is highlighted in bold face; positions where Pro residues were exchanged to Ala are underlined; the cysteine residues used for labeling are shown in red. The sequences correspond to UniprotKB entries Q92793 (CBP_HUMAN), residues 2060-2216 (NCBD) and Q9Y6Q9 (NCOA3_HUMAN), residues 1023-1093 (ACTR).

Variant	Sequence
NCBD-Avi	MAGLNDIFEAQKIEWHEGSMGS<br>GSC **P**NRSISPSAL QDLLRTLKSP SSPQQQQQVL NILKSNPQLM AAFIKQRTAK YVANQPGMQ GGPR
NCBD-Avi P20A	MAGLNDIFEAQKIEWHEGSMGS<br>GSC **P**NRSISPSAL QDLLRTLKSA SSPQQQQQVL NILKSNPQLM AAFIKQRTAK YVANQPGMQ GGPR
NCBD wt	GPC **P**NRSISPSAL QDLLRTLKSP SSPQQQQQVL NILKSNPQLM AAFIKQRTAK YVANQPGMQ
NCBD P20A	GPC **P**NRSISPSAL QDLLRTLKSA SSPQQQQQVL NILKSNPQLM AAFIKQRTAK YVANQPGMQC
NCBD P23A	GPC **P**NRSISPSAL QDLLRTLKSP SSAQQQQQVL NILKSNPQLM AAFIKQRTAK YVANQPGMQC
NCBD P20/23A	GPC **P**NRSISPSAL QDLLRTLKSA SSAQQQQQVL NILKSNPQLM AAFIKQRTAK YVANQPGMQC
NCBD P7/37A	GPC **P**NRSISASAL QDLLRTLKSP SSPQQQQQVL NILKSNAQLM AAFIKQRTAK YVANQPGMQC
NCBD P7/20/23A	GPC **P**NRSISASAL QDLLRTLKSA SSAQQQQQVL NILKSNPQLM AAFIKQRTAK YVANQPGMQC
NCBD P20/23/37A	GPC **P**NRSISPSAL QDLLRTLKSA SSAQQQQQVL NILKSNAQLM AAFIKQRTAK YVANQPGMQC
NCBD P7/23/37A	GPC **P**NRSISASAL QDLLRTLKSP SSAQQQQQVL NILKSNAQLM AAFIKQRTAK YVANQPGMQC
NCBD P7/20/23/37A	GPC **P**NRSISASAL QDLLRTLKSA SSAQQQQQVL NILKSNAQLM AAFIKQRTAK YVANQPGMQC
ACTR-Avi	MAGLNDIFEAQKIEWHEGSMGS<br>GS GTQNRPLLRN SLDDLVGPPS NLEGQSDERA LLDQLHTLLS NTDATGLEEI DRALGIPELV NQGQALEPKQ DC GGPR
ACTR	GP GTQNRPLLRN SLDDLVGPPS NLEGQSDERA LLDQLHTLLS NTDATGLEEI DRALGIPELV NQGQALEPKQ DC

Supplementary Table 4. Primers used in the construction of NCDB and ACTR variants. All primers were obtained at Microsynth.

Primer	Sequence
NCBD_P20A_fw	GCT GAA ATC GGC ATC TTC ACC GCA ACA GCA ACA GC
NCBD_P20A_rev	GGT GAA GAT GCC GAT TTC AGC GTC CTC AGA AGG
NCBD_P23A_fw	CCA TCT TCA GCG CAA CAG CAA CAG CAA GTT CTT AAC
NCBD_P23A_rev	GCT GTT GCG CTG AAG ATG GCG ATT TCA GCG TCC
NCBD_P20A_P23A_fw	GCA TCT TCA GCG CAA CAG CAA CAG CAA GTT CTT AAC
NCBD_P20A_P23A_rev	GCT GTT GCG CTG AAG ATG CCG ATT TCA GCG TCC
NCBD_P7A_fw	GTA TAA GCG CGT CCG CCC TGC AGG ACC
NCBD_P7A_rev	GGC GGA CGC GCT TAT ACT ACG GTT CGG
NCBD_P37A_fw	AAA GCA ATG CTC AAC TGA TGG CGG CTT TC
NCBD_P37A_rev	CAG TTG AGC ATT GCT TTT AAG AAT GTT AAG
ACTR Cys3rem_fw	CAG GGC CCT GGT ACC CAG AAT CGC CCG TTG C
ACTR Cys3rem_rev	CTG GGT ACC AGG GCC CTG GAA TAA CAC CTC
ACTR_Avi Cys3rem_fw	GGG ATC CGG CAG CGG TAC CCA GAA TCG CCC GTT GCT GCG
ACTR_Avi Cys3rem_rev	CTG GGT ACC GCT GCC GGA TCC CAT GGA ACC TTC GTG CC

Supplementary References

1 Demarest, S. J. *et al.* Mutual synergistic folding in recruitment of CBP/p300 by p160 nuclear receptor coactivators. *Nature* **415**, 549-553, (2002).

2 Humphrey, W., Dalke, A. & Schulten, K. VMD: Visual molecular dynamics. *J. Mol. Graph.* **14**, 33-38, (1996).

3 Schuster-Bockler, B., Schultz, J. & Rahmann, S. HMM Logos for visualization of protein families. *BMC Bioinformatics* **5**, 7, (2004).

4 Punta, M. *et al.* The Pfam protein families database. *Nucleic Acids Research* **40**, D290-301, (2012).
